# 7-Bromo-1-methyl­sulfinyl-2-phenyl­naphtho[2,1-*b*]furan

**DOI:** 10.1107/S1600536809028165

**Published:** 2009-07-22

**Authors:** Hong Dae Choi, Pil Ja Seo, Byeng Wha Son, Uk Lee

**Affiliations:** a Department of Chemistry, Dongeui University, San 24 Kaya-dong Busanjin-gu, Busan 614-714, Republic of Korea; b Department of Chemistry, Pukyong National University, 599-1 Daeyeon 3-dong, Nam-gu, Busan 608-737, Republic of Korea

## Abstract

In the title compound, C_19_H_13_BrO_2_S, the O atom and the methyl group of the methyl­sulfinyl substituent lie on opposite sides of the plane of the naphthofuran unit. The phenyl ring is rotated out of the naphthofuran plane, making a dihedral angle of 42.2 (1)°. The crystal structure is stabilized by two inter­molecular C—H⋯π inter­actions, and by non-classical inter­molecular C—H⋯O and C—H⋯Br hydrogen bonds.

## Related literature

For the crystal structures of similar 2-phenyl­naphtho[2,1-*b*]furan derivatives, see: Choi *et al.* (2009*a* ▶,*b* ▶). For details of the biological and pharmacological activity of naphthofuran compounds, see: Goel & Dixit (2004 ▶); Hagiwara *et al.* (1999 ▶); Piloto *et al.* (2005 ▶).
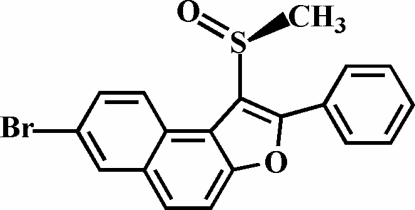


## Experimental

### 

#### Crystal data

C_19_H_13_BrO_2_S
*M* *_r_* = 385.26
Monoclinic, 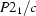

*a* = 6.0007 (4) Å
*b* = 22.699 (2) Å
*c* = 11.2151 (8) Å
β = 91.267 (1)°
*V* = 1527.2 (2) Å^3^
*Z* = 4
Mo *K*α radiation
μ = 2.84 mm^−1^
*T* = 273 K
0.25 × 0.12 × 0.10 mm

#### Data collection

Bruker SMART CCD diffractometer
Absorption correction: multi-scan (*SADABS*; Sheldrick, 1999 ▶) *T*_min_ = 0.538, *T*_max_ = 0.765
13412 measured reflections
3476 independent reflections
2280 reflections with *I* > 2σ(*I*)
*R*_int_ = 0.056

#### Refinement

*R*[*F* ^2^ > 2σ(*F* ^2^)] = 0.039
*wR*(*F* ^2^) = 0.091
*S* = 1.09
3476 reflections
209 parameters
H-atom parameters constrained
Δρ_max_ = 0.56 e Å^−3^
Δρ_min_ = −0.49 e Å^−3^

### 

Data collection: *SMART* (Bruker, 2001 ▶); cell refinement: *SAINT* (Bruker, 2001 ▶); data reduction: *SAINT*; program(s) used to solve structure: *SHELXS97* (Sheldrick, 2008 ▶); program(s) used to refine structure: *SHELXL97* (Sheldrick, 2008 ▶); molecular graphics: *ORTEP-3* (Farrugia, 1997 ▶) and *DIAMOND* (Brandenburg, 1998 ▶); software used to prepare material for publication: *SHELXL97*.

## Supplementary Material

Crystal structure: contains datablocks global, I. DOI: 10.1107/S1600536809028165/rk2156sup1.cif

Structure factors: contains datablocks I. DOI: 10.1107/S1600536809028165/rk2156Isup2.hkl

Additional supplementary materials:  crystallographic information; 3D view; checkCIF report

## Figures and Tables

**Table 1:** Hydrogen-bond geometry (Å, °)

*D*—H⋯*A*	*D*—H	H⋯*A*	*D*⋯*A*	*D*—H⋯*A*
C14—H14⋯*Cg*1^i^	0.93	2.70	3.377 (4)	131
C19—H19*B*⋯*Cg*2^ii^	0.96	2.99	3.497 (4)	114
C18—H18⋯O2^iii^	0.93	2.54	3.283 (4)	137
C16—H16⋯Br^iv^	0.93	2.97	3.823 (4)	153

Symmetry codes: (i) 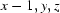; (ii) 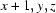; (iii) 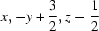; (iv) 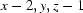. *Cg*1 and *Cg*2 are the centroids of the C2/C3/C8–C11 benzene and C13–C18 benzene rings, respectively.

**Fig. 1.**
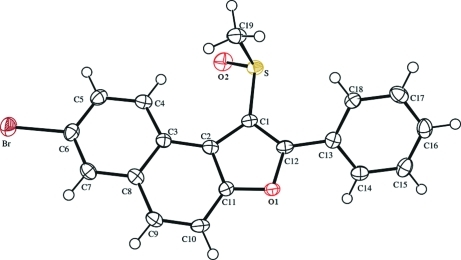
The molecular structure of the title compound with the atom numbering scheme. Displacement ellipsoids are drawn at the 30% probability level. H atoms are presented as a small spheres of arbitrary radius.

**Fig. 2.**
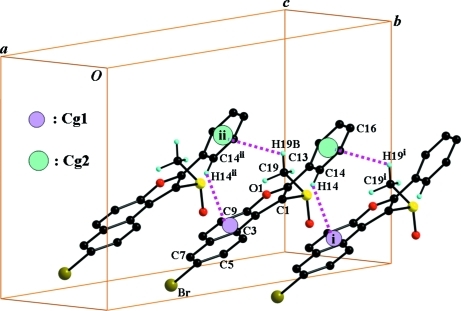
The C–H···π interactions (dotted lines) in the crystal structure of title compound. Cg denotes the ring centroids. Symmetry codes: (i) x-1, y, z; (ii) x+1, y, z.

**Fig. 3.**
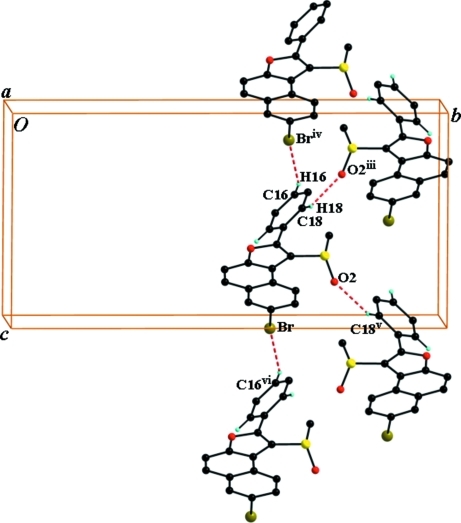
The C–H···O and C–H···Br interactions (dotted lines) in the title compound. Symmetry codes: (iii) x, -y+3/2, z-1/2; (iv) x-2, y, z-1; (v) x, -y+3/2, z+1/2; (vi) x+2, y, z+1.

## supplementary crystallographic information

### Comment

Molecules containing naphthofuran skeleton have attracted considerable interest in view of their biological and pharmacological activities (Goel & Dixit, 2004; Hagiwara *et al.*, 1999; Piloto *et al*., 2005). This work is related to our communications on the synthesis and structures of 2-phenylnaphtho[2,1-b]furan analogues, *viz*. 2-phenyl-1-(phenylsulfinyl)naphtho[2,1-b]furan (Choi *et al.*, 2009*a*) and 7-bromo-2-phenyl-1-(phenylsulfinyl)naphtho[2,1-b]furan (Choi *et al.*, 2009*b*). Now we present the crystal structure of the title compound (I) (Fig. 1).

The naphthofuran unit is essentially planar, with a mean deviation of 0.045 (3)Å from the least-squares plane defined by the thirteen constituent atoms. The dihedral angle in I formed by the plane of the naphthofuran ring and the plane of 2-phenyl ring is 42.2 (1)°. The molecular packing (Fig. 2) is stabilized by two intermolecular C–H···π interactions; the first between an H atom of the 2-phenyl ring and the central benzene ring of the naphthofuran fragment (C14–H14···Cg1^i^), the second between the methyl H atom of the methylsulfinyl substituent and the phenyl ring (C19–H19B···Cg2^ii^), respectively (Table 1 and Fig. 2; Cg1 and Cg2 are the centroids of the C2/C3/C8/C9/C10/C11 benzene and C13-C18 benzene rings). Symmetry codes: (i) *x*-1, *y*, *z*; (ii) *x*+1, *y*, *z*. In addition, weak non-classical intermolecular C–H···O and C–H···Br hydrogen bonds in the structure were observed (Table 1 and Fig. 3).

### Experimental

The 77% 3-chloroperoxybenzoic acid (157 mg, 0.7 mmol) was added in small portions to a stirred solution of 7-bromo-1-methylsulfanyl-2-phenylnaphtho[2,1-b]furan (258 mg, 0.7 mmol) in dichloromethane (40 mL) at 273 K. After being stirred at room temperature for 4h, the mixture was washed with saturated sodium bicarbonate solution and the organic layer was separated, dried over magnesium sulfate, filtered and concentrated in vacuum. The residue was purified by column chromatography (hexane-ethyl acetate, 1 : 2 v/v) to afford the title compound as a colorless solid [yield 78%, m.p. 503-504 K; *R*_f_ = 0.72 (hexane-ethyl acetate, 1 : 2 v/v)]. Single crystals suitable for X-ray diffraction were prepared by slow evaporation of a solution of the title compound in dichloromethane at room temperature.

### Refinement

All H atoms were geometrically positioned and refined using a riding model, with C–H = 0.93 Å for the aryl and 0.96 Å for the methyl H atoms. *U*_iso_(H) = 1.2*U*_eq_(C) for the aryl H atoms and 1.5*U*_eq_(C) for methyl H atoms.

### Crystal data

**Table:**

C_19_H_13_BrO_2_S	*F*(000) = 776
*M**_r_* = 385.26	*D*_x_ = 1.676 Mg m^−^^3^
Monoclinic, *P*2_1_/*c*	Mo *K*α radiation, λ = 0.71073 Å
Hall symbol: -P 2ybc	Cell parameters from 3117 reflections
*a* = 6.0007 (4) Å	θ = 2.6–27.3°
*b* = 22.699 (2) Å	µ = 2.84 mm^−^^1^
*c* = 11.2151 (8) Å	*T* = 273 K
β = 91.267 (1)°	Block, colourless
*V* = 1527.2 (2) Å^3^	0.25 × 0.12 × 0.10 mm
*Z* = 4	

### Data collection

**Table:**

Bruker SMART CCD diffractometer	3476 independent reflections
Radiation source: fine-focus sealed tube	2280 reflections with *I* > 2σ(*I*)
graphite	*R*_int_ = 0.056
Detector resolution: 10.0 pixels mm^-1^	θ_max_ = 27.5°, θ_min_ = 1.8°
φ and ω scans	*h* = −7→7
Absorption correction: multi-scan (*SADABS*; Sheldrick, 1999)	*k* = −29→29
*T*_min_ = 0.538, *T*_max_ = 0.765	*l* = −14→14
13412 measured reflections	

### Refinement

**Table:**

Refinement on *F*^2^	Primary atom site location: structure-invariant direct methods
Least-squares matrix: full	Secondary atom site location: difference Fourier map
*R*[*F*^2^ > 2σ(*F*^2^)] = 0.039	Hydrogen site location: difference Fourier map
*wR*(*F*^2^) = 0.091	H-atom parameters constrained
*S* = 1.09	*w* = 1/[σ^2^(*F*_o_^2^) + (0.0234*P*)^2^ + 2.3626*P*] where *P* = (*F*_o_^2^ + 2*F*_c_^2^)/3
3476 reflections	(Δ/σ)_max_ = 0.002
209 parameters	Δρ_max_ = 0.56 e Å^−^^3^
0 restraints	Δρ_min_ = −0.49 e Å^−^^3^

### Special details

**Table:**

Geometry. All s.u.'s (except the s.u. in the dihedral angle between two l.s. planes) are estimated using the full covariance matrix. The cell s.u.'s are taken into account individually in the estimation of s.u.'s in distances, angles and torsion angles; correlations between s.u.'s in cell parameters are only used when they are defined by crystal symmetry. An approximate (isotropic) treatment of cell s.u.'s is used for estimating s.u.'s involving l.s. planes.
Refinement. Refinement of F^2^ against ALL reflections. The weighted R-factor wR and goodness of fit S are based on F^2^, conventional R-factors R are based on F, with F set to zero for negative F^2^. The threshold expression of F^2^ > 2σ(F^2^) is used only for calculating R-factors(gt) etc. and is not relevant to the choice of reflections for refinement. R-factors based on F^2^ are statistically about twice as large as those based on F, and R-factors based on ALL data will be even larger.

### Fractional atomic coordinates and isotropic or equivalent isotropic displacement parameters (Å^2^)

**Table:**

	*x*	*y*	*z*	*U*_iso_*/*U*_eq_	
Br	1.04085 (7)	0.614541 (18)	1.06514 (4)	0.03289 (13)	
S	0.04668 (16)	0.71912 (4)	0.66608 (8)	0.0231 (2)	
O1	−0.0349 (4)	0.54760 (10)	0.6267 (2)	0.0208 (5)	
O2	0.0802 (4)	0.74221 (11)	0.7894 (2)	0.0316 (7)	
C1	0.0664 (6)	0.64129 (15)	0.6686 (3)	0.0191 (7)	
C2	0.2146 (6)	0.60190 (15)	0.7355 (3)	0.0195 (8)	
C3	0.4067 (6)	0.60777 (15)	0.8130 (3)	0.0194 (7)	
C4	0.4927 (6)	0.66186 (16)	0.8559 (3)	0.0234 (8)	
H4	0.4210	0.6967	0.8343	0.028*	
C5	0.6794 (6)	0.66431 (17)	0.9287 (3)	0.0255 (9)	
H5	0.7343	0.7003	0.9560	0.031*	
C6	0.7857 (6)	0.61148 (17)	0.9610 (3)	0.0243 (8)	
C7	0.7101 (6)	0.55824 (16)	0.9228 (3)	0.0237 (8)	
H7	0.7854	0.5241	0.9455	0.028*	
C8	0.5162 (6)	0.55461 (16)	0.8484 (3)	0.0210 (8)	
C9	0.4331 (6)	0.49849 (16)	0.8115 (3)	0.0230 (8)	
H9	0.5084	0.4646	0.8357	0.028*	
C10	0.2455 (6)	0.49362 (15)	0.7415 (3)	0.0233 (8)	
H10	0.1883	0.4571	0.7193	0.028*	
C11	0.1437 (6)	0.54571 (15)	0.7050 (3)	0.0196 (8)	
C12	−0.0772 (6)	0.60640 (15)	0.6050 (3)	0.0199 (8)	
C13	−0.2527 (6)	0.61732 (16)	0.5143 (3)	0.0206 (7)	
C14	−0.4437 (6)	0.58201 (15)	0.5112 (3)	0.0226 (8)	
H14	−0.4595	0.5521	0.5671	0.027*	
C15	−0.6085 (6)	0.59122 (17)	0.4259 (3)	0.0273 (9)	
H15	−0.7349	0.5675	0.4241	0.033*	
C16	−0.5855 (7)	0.63605 (17)	0.3422 (3)	0.0286 (9)	
H16	−0.6985	0.6430	0.2859	0.034*	
C17	−0.3945 (7)	0.67033 (16)	0.3429 (3)	0.0284 (9)	
H17	−0.3785	0.6998	0.2861	0.034*	
C18	−0.2277 (6)	0.66085 (15)	0.4275 (3)	0.0229 (8)	
H18	−0.0985	0.6835	0.4267	0.028*	
C19	0.2979 (7)	0.73401 (16)	0.5874 (3)	0.0284 (9)	
H19A	0.3314	0.7753	0.5921	0.043*	
H19B	0.2779	0.7228	0.5053	0.043*	
H19C	0.4187	0.7119	0.6226	0.043*	

### Atomic displacement parameters (Å^2^)

**Table:**

	*U*^11^	*U*^22^	*U*^33^	*U*^12^	*U*^13^	*U*^23^
Br	0.0306 (2)	0.0375 (2)	0.0302 (2)	−0.0047 (2)	−0.00788 (15)	0.0030 (2)
S	0.0258 (5)	0.0177 (5)	0.0260 (5)	0.0017 (4)	0.0018 (4)	−0.0027 (4)
O1	0.0258 (14)	0.0156 (12)	0.0210 (13)	−0.0033 (10)	0.0000 (11)	0.0003 (10)
O2	0.0401 (17)	0.0287 (15)	0.0262 (15)	0.0017 (13)	0.0037 (12)	−0.0113 (12)
C1	0.0228 (19)	0.0155 (17)	0.0191 (18)	−0.0026 (15)	0.0037 (14)	−0.0009 (14)
C2	0.0244 (19)	0.0190 (19)	0.0154 (17)	−0.0003 (15)	0.0047 (14)	0.0001 (14)
C3	0.0215 (18)	0.0202 (18)	0.0168 (17)	−0.0005 (15)	0.0030 (14)	0.0011 (15)
C4	0.028 (2)	0.0196 (19)	0.022 (2)	−0.0003 (16)	0.0027 (16)	0.0015 (15)
C5	0.028 (2)	0.026 (2)	0.022 (2)	−0.0066 (17)	0.0003 (16)	−0.0022 (16)
C6	0.0241 (19)	0.031 (2)	0.0177 (17)	−0.0044 (18)	−0.0007 (14)	0.0000 (17)
C7	0.027 (2)	0.025 (2)	0.0191 (18)	0.0043 (17)	0.0010 (15)	0.0022 (16)
C8	0.026 (2)	0.0229 (19)	0.0148 (17)	0.0037 (16)	0.0037 (15)	0.0005 (15)
C9	0.030 (2)	0.0195 (19)	0.0193 (19)	0.0040 (16)	0.0005 (16)	0.0020 (15)
C10	0.036 (2)	0.0162 (18)	0.0184 (19)	−0.0054 (16)	0.0037 (16)	−0.0006 (15)
C11	0.0215 (19)	0.0195 (18)	0.0178 (18)	−0.0020 (15)	0.0018 (15)	−0.0009 (14)
C12	0.0227 (19)	0.0163 (19)	0.0210 (18)	−0.0006 (15)	0.0060 (14)	−0.0019 (14)
C13	0.0211 (18)	0.0220 (18)	0.0188 (17)	0.0031 (16)	0.0041 (14)	−0.0055 (16)
C14	0.026 (2)	0.0186 (19)	0.0236 (19)	0.0014 (16)	0.0055 (16)	−0.0032 (15)
C15	0.021 (2)	0.032 (2)	0.029 (2)	0.0007 (16)	−0.0001 (16)	−0.0096 (17)
C16	0.032 (2)	0.030 (2)	0.024 (2)	0.0073 (18)	−0.0055 (17)	−0.0074 (17)
C17	0.040 (2)	0.023 (2)	0.022 (2)	0.0049 (18)	0.0018 (18)	0.0003 (16)
C18	0.023 (2)	0.0210 (19)	0.025 (2)	−0.0013 (16)	0.0005 (16)	−0.0022 (15)
C19	0.037 (2)	0.022 (2)	0.026 (2)	−0.0037 (18)	0.0034 (17)	−0.0001 (16)

### Geometric parameters (Å, °)

**Table:**

Br—C6	1.906 (3)	C9—C10	1.362 (5)
S—O2	1.489 (3)	C9—H9	0.9300
S—C1	1.771 (3)	C10—C11	1.389 (5)
S—C19	1.796 (4)	C10—H10	0.9300
O1—C11	1.371 (4)	C12—C13	1.469 (5)
O1—C12	1.379 (4)	C13—C18	1.398 (5)
C1—C12	1.360 (5)	C13—C14	1.398 (5)
C1—C2	1.457 (5)	C14—C15	1.377 (5)
C2—C11	1.385 (5)	C14—H14	0.9300
C2—C3	1.434 (5)	C15—C16	1.393 (5)
C3—C4	1.412 (5)	C15—H15	0.9300
C3—C8	1.426 (5)	C16—C17	1.385 (5)
C4—C5	1.373 (5)	C16—H16	0.9300
C4—H4	0.9300	C17—C18	1.381 (5)
C5—C6	1.402 (5)	C17—H17	0.9300
C5—H5	0.9300	C18—H18	0.9300
C6—C7	1.357 (5)	C19—H19A	0.9600
C7—C8	1.420 (5)	C19—H19B	0.9600
C7—H7	0.9300	C19—H19C	0.9600
C8—C9	1.426 (5)		
			
O2—S—C1	109.20 (16)	C9—C10—H10	121.5
O2—S—C19	107.03 (17)	C11—C10—H10	121.5
C1—S—C19	97.99 (17)	O1—C11—C2	111.2 (3)
C11—O1—C12	106.3 (3)	O1—C11—C10	123.2 (3)
C12—C1—C2	106.6 (3)	C2—C11—C10	125.5 (3)
C12—C1—S	122.1 (3)	C1—C12—O1	111.1 (3)
C2—C1—S	131.3 (3)	C1—C12—C13	134.5 (3)
C11—C2—C3	118.3 (3)	O1—C12—C13	114.3 (3)
C11—C2—C1	104.9 (3)	C18—C13—C14	119.2 (3)
C3—C2—C1	136.7 (3)	C18—C13—C12	121.0 (3)
C4—C3—C8	118.6 (3)	C14—C13—C12	119.7 (3)
C4—C3—C2	124.8 (3)	C15—C14—C13	120.4 (3)
C8—C3—C2	116.7 (3)	C15—C14—H14	119.8
C5—C4—C3	121.7 (3)	C13—C14—H14	119.8
C5—C4—H4	119.2	C14—C15—C16	119.9 (4)
C3—C4—H4	119.2	C14—C15—H15	120.0
C4—C5—C6	118.7 (3)	C16—C15—H15	120.0
C4—C5—H5	120.7	C17—C16—C15	120.1 (4)
C6—C5—H5	120.7	C17—C16—H16	120.0
C7—C6—C5	122.2 (3)	C15—C16—H16	120.0
C7—C6—Br	119.0 (3)	C18—C17—C16	120.2 (4)
C5—C6—Br	118.8 (3)	C18—C17—H17	119.9
C6—C7—C8	120.1 (3)	C16—C17—H17	119.9
C6—C7—H7	120.0	C17—C18—C13	120.1 (3)
C8—C7—H7	120.0	C17—C18—H18	119.9
C7—C8—C9	119.9 (3)	C13—C18—H18	119.9
C7—C8—C3	118.8 (3)	S—C19—H19A	109.5
C9—C8—C3	121.3 (3)	S—C19—H19B	109.5
C10—C9—C8	121.2 (3)	H19A—C19—H19B	109.5
C10—C9—H9	119.4	S—C19—H19C	109.5
C8—C9—H9	119.4	H19A—C19—H19C	109.5
C9—C10—C11	117.0 (3)	H19B—C19—H19C	109.5
			
O2—S—C1—C12	137.3 (3)	C8—C9—C10—C11	1.9 (5)
C19—S—C1—C12	−111.5 (3)	C12—O1—C11—C2	1.0 (4)
O2—S—C1—C2	−39.4 (4)	C12—O1—C11—C10	−175.3 (3)
C19—S—C1—C2	71.8 (4)	C3—C2—C11—O1	−177.3 (3)
C12—C1—C2—C11	−0.1 (4)	C1—C2—C11—O1	−0.6 (4)
S—C1—C2—C11	177.1 (3)	C3—C2—C11—C10	−1.1 (5)
C12—C1—C2—C3	175.8 (4)	C1—C2—C11—C10	175.6 (3)
S—C1—C2—C3	−7.1 (6)	C9—C10—C11—O1	174.5 (3)
C11—C2—C3—C4	−177.2 (3)	C9—C10—C11—C2	−1.3 (5)
C1—C2—C3—C4	7.4 (7)	C2—C1—C12—O1	0.7 (4)
C11—C2—C3—C8	2.7 (5)	S—C1—C12—O1	−176.8 (2)
C1—C2—C3—C8	−172.7 (4)	C2—C1—C12—C13	−174.3 (4)
C8—C3—C4—C5	1.0 (5)	S—C1—C12—C13	8.3 (6)
C2—C3—C4—C5	−179.1 (3)	C11—O1—C12—C1	−1.0 (4)
C3—C4—C5—C6	−0.2 (6)	C11—O1—C12—C13	175.0 (3)
C4—C5—C6—C7	0.0 (6)	C1—C12—C13—C18	37.7 (6)
C4—C5—C6—Br	−178.5 (3)	O1—C12—C13—C18	−137.1 (3)
C5—C6—C7—C8	−0.5 (5)	C1—C12—C13—C14	−145.2 (4)
Br—C6—C7—C8	178.0 (3)	O1—C12—C13—C14	40.0 (4)
C6—C7—C8—C9	−178.0 (3)	C18—C13—C14—C15	−2.0 (5)
C6—C7—C8—C3	1.3 (5)	C12—C13—C14—C15	−179.2 (3)
C4—C3—C8—C7	−1.5 (5)	C13—C14—C15—C16	−0.2 (5)
C2—C3—C8—C7	178.6 (3)	C14—C15—C16—C17	1.7 (6)
C4—C3—C8—C9	177.8 (3)	C15—C16—C17—C18	−1.0 (6)
C2—C3—C8—C9	−2.1 (5)	C16—C17—C18—C13	−1.2 (6)
C7—C8—C9—C10	179.0 (3)	C14—C13—C18—C17	2.7 (5)
C3—C8—C9—C10	−0.3 (5)	C12—C13—C18—C17	179.9 (3)

### Hydrogen-bond geometry (Å, °)

**Table:**

*D*—H···*A*	*D*—H	H···*A*	*D*···*A*	*D*—H···*A*
C14—H14···Cg1^i^	0.93	2.70	3.377 (4)	131
C19—H19B···Cg2^ii^	0.96	2.99	3.497 (4)	114
C18—H18···O2^iii^	0.93	2.54	3.283 (4)	137
C16—H16···Br^iv^	0.93	2.97	3.823 (4)	153

Symmetry codes: (i) *x*−1, *y*, *z*; (ii) *x*+1, *y*, *z*; (iii) *x*, −*y*+3/2, *z*−1/2; (iv) *x*−2, *y*, *z*−1.
